# Information and Innovation: A Natural Combination for Health Sciences Libraries

**DOI:** 10.5195/jmla.2018.508

**Published:** 2018-10-01

**Authors:** Martha F. Earl

**Affiliations:** Preston Medical Library, Graduate School of Medicine, University of Tennessee, Knoxville, TN

As the editors state in the preface, “With the decline of clinical income and research dollars, many academic health sciences centers are looking toward innovative product development as their new income source.” As partners with those innovators, librarians can offer resources and services for research, evidence, training, dissemination venues, and collaborative physical spaces with state-of-the-art equipment, including 3D printers, models, scanners, and video monitors.

Public libraries have long partnered with their business and educational community members to offer makerspaces and to spark innovative and profitable ventures. This book addresses how academic health sciences libraries can meet those same needs to benefit their institutions, the innovators, and librarians themselves by strengthening the role of librarians in promoting innovation in their institutions.

The editors, Jean P. Shipman, AHIP, FMLA, and Barbara A. Ulmer, represent the two aspects in the title. Shipman until recently directed the University of Utah Eccles Health Sciences Library, where she earned her reputation as a leader of innovation in the field of academic health sciences librarianship. She shares her well-planned and executed projects and partnerships. Ulmer has a background in financial and technologically innovative process analysis. Their expertise will benefit those librarians who seek the why and how to take the next step into the future with technology. Another leader in health sciences libraries, M.J. Tooey, AHIP, FMLA, is a chapter contributor.

The text includes sixteen chapters. The first chapter by Temple University’s Joseph Lucia provides an historical overview. In the second chapter, Shipman and colleagues describe the development cycle of innovation, based on evidence and key knowledge. Chapters 3 through 8 focus on libraries as innovation spaces with case studies of how three different health sciences libraries proactively sought involvement with campus innovators and redesigned static library spaces to be more dynamic and productive service areas.

Chapters 9 and 10 illustrate how creators of digital medical therapeutic games seek and use information and how an innovation librarian can meet those needs. In chapter 10, a gaming competition involving general undergraduate students; graduate, medical, and law campus students; and their librarian team demonstrates the value of collaboration in cross-disciplinary studies and game development. In chapter 11, a library director and an innovation librarian describe the benefits and challenges of working with students, faculty, and community industry mentors. Professional development for the innovation librarian must be part of the plan to build this type of service.

Chapters 12 through 15 focus on specific projects at the University of Utah Eccles Health Sciences Library. Chapter 12 details the use of technology in developing games and apps to change patient education and consumer behavior. Chapter 13 describes the development of the e-channel, a web-based multimedia portal to capture and document productivity nationally and internationally while contributing to university promotion and tenure portfolios. Chapter 14 outlines the step-by-step development of NOVEL, another multimedia product, resulting this time from the partnership between the Eccles Health Sciences Library and a professional society. Chapter 15 describes the development and use of the Innovation Vault, an indexed database of videos designed as an easy-to-use discovery tool to educate those who are interested in innovation techniques and products.

In chapter 16, the editors explore the potential future of the partnership between libraries and innovators, emphasizing learning about what those collaborations look like and how librarians can be prepared to seize such opportunities. Various chapters address the funding of these projects and how collaborative efforts can mean an increased funding stream for both institutions and their libraries.

The book includes a foreword by the head of the innovation center at the University of Utah, a preface by the editors that includes citations with a list of websites commonly used in the book, a list of twenty figures and illustrations, and a list of four tables. Each chapter is footnoted with citations listed as notes as the end of the chapter. The index is cross-referenced.

Another valuable addition to this book is the well-annotated section, “About the Editors and Contributors,” which helps readers understand the relationships between the numerous collaborators and contributors, who include professionals in libraries, medicine, education, engineering, and technology.

This publication is one of a series of Medical Library Association (MLA) books chosen by the MLA Books Panel to contribute to best practices for health sciences librarians. This book can be used by educators, instructional designers, and information technology professionals in academic health sciences and hospital settings, as well as by their library colleagues.

This book is recommended for academic health sciences libraries and academic teaching hospital libraries with funding and staff to support innovation. University libraries supporting health sciences, technology, and information sciences programs may also find it valuable.

